# A Novel NIR-FRET Biosensor for Reporting PS/γ-Secretase Activity in Live Cells

**DOI:** 10.3390/s20215980

**Published:** 2020-10-22

**Authors:** Mei CQ Houser, Steven S Hou, Florian Perrin, Yuliia Turchyna, Brian J Bacskai, Oksana Berezovska, Masato Maesako

**Affiliations:** Alzheimer Research Unit, MassGeneral Institute for Neurodegenerative Disease, Massachusetts General Hospital, Harvard Medical School, 114, 16th street, Charlestown, MA 02129, USA; mhouser@mgh.harvard.edu (M.C.Q.H.); shou@partners.org (S.S.H.); fperrin@mgh.harvard.edu (F.P.); yturchyna@mgh.harvard.edu (Y.T.); bbacskai@mgh.harvard.edu (B.J.B.); oberezovska@mgh.harvard.edu (O.B.)

**Keywords:** Presenilin (PS)/γ-secretase, FRET biosensor, near-infrared (NIR), miRFP670 and 720

## Abstract

Presenilin (PS)/γ-secretase plays a pivotal role in essential cellular events via proteolytic processing of transmembrane proteins that include APP and Notch receptors. However, how PS/γ-secretase activity is spatiotemporally regulated by other molecular and cellular factors and how the changes in PS/γ-secretase activity influence signaling pathways in live cells are poorly understood. These questions could be addressed by engineering a new tool that enables multiplexed imaging of PS/γ-secretase activity and additional cellular events in real-time. Here, we report the development of a near-infrared (NIR) FRET-based PS/γ-secretase biosensor, C99 720-670 probe, which incorporates an immediate PS/γ-secretase substrate APP C99 with miRFP670 and miRFP720 as the donor and acceptor fluorescent proteins, respectively. Extensive validation demonstrates that the C99 720-670 biosensor enables quantitative monitoring of endogenous PS/γ-secretase activity on a cell-by-cell basis in live cells (720/670 ratio: 2.47 ± 0.66 (vehicle) vs. 3.02 ± 1.17 (DAPT), ** *p* < 0.01). Importantly, the C99 720-670 and the previously developed APP C99 YPet-Turquoise-GL (C99 Y-T) biosensors simultaneously report PS/γ-secretase activity. This evidences the compatibility of the C99 720-670 biosensor with cyan (CFP)-yellow fluorescent protein (YFP)-based FRET biosensors for reporting other essential cellular events. Multiplexed imaging using the novel NIR biosensor C99 720-670 would open a new avenue to better understand the regulation and consequences of changes in PS/γ-secretase activity.

## 1. Introduction

Presenilin (PS)/γ-secretase is a membrane-embedded protease responsible for proteolytic processing of a wide variety of membrane-associated proteins that include the Amyloid precursor protein (APP) and Notch receptors [1,2,3]. Four molecules, namely PS, Nicastrin, Pen2 and Aph1, compose a PS/γ-secretase complex [4,5,6,7,8]. PS serves as the catalytic core [9,10,11] and has two isoforms PS1 and PS2 [12,13]. PS/γ-secretase is ubiquitously expressed throughout the body and plays a significant role in regulating multiple essential functions in a broad range of cell types. For instance, the PS1/2 knockout mice are lethal due to abnormal skeletal and vasculature formation, impaired neurogenesis and neuronal survival, highlighting its significant roles during development [14,15]. Additionally, mutations on the genes encoding PS result in familial Alzheimer’s disease (AD) [12,13], Frontotemporal dementia (FTD) [16,17] or Acne Inversa (an inflammatory skin disease) [18,19,20], implicating that changes in PS/γ-secretase activity could be responsible for the development of sporadic cases of the diseases.

To better understand how PS/γ-secretase activity is spatiotemporally regulated in live cells, we recently developed and validated a Förster resonance energy transfer (FRET) biosensor, the so-called C99 Y-T biosensor, that contains Turquoise-GL and YPet as the donor and acceptor fluorescent proteins, respectively [21]. By using the C99 Y-T biosensor and quantitative monitoring of PS/γ-secretase activity in live/intact cells overtime on a cell-by-cell basis, we uncovered heterogeneous regulation of PS/γ-secretase activity among live neurons [21]. Multiple proteins such as calcium binding or hypoxia related proteins are reported to form complexes with PS/γ-secretase and potentially modulate PS/γ-secretase activity [22,23,24]. Moreover, the high number of serine and threonine residues within PS suggests that its phosphorylated or dephosphorylated state can modulate the enzymatic function of PS/γ-secretase [25,26,27,28,29,30,31,32]. These findings imply that PS/γ-secretase activity could be affected by other essential cell factors in live/intact cells.

This study aimed to develop and validate a new near-infrared (NIR) PS/γ-secretase activity biosensor that enables simultaneous recording of PS/γ-secretase activity and other cellular signaling events. For this, we utilized two recently engineered technologies: the C99 Y-T FRET biosensor for monitoring PS/γ-secretase activity [21] and NIR fluorescent proteins miRFP670 and miRFP720 [33,34]. This resulted in the development of the NIR C99 miRFP720-miRFP670 (C99 720-670) biosensor that can quantitatively report PS/γ-secretase activity in live cells. Here, we show that the C99 720-670 biosensor is processed by endogenous PS/γ-secretase, can distinguish PS/γ-secretase activity in individual cells, reports PS/γ-secretase activity over time and, importantly, is compatible with the cyan (CFP)-yellow fluorescent protein (YFP)-based biosensor. The novel C99 720-670 biosensor could break new ground for a better understanding of the regulation and consequence of changes in PS/γ-secretase activity in live cells.

## 2. Materials and Methods

### 2.1. Plasmid DNA

The cDNA of miRFP670 and iRFP720 were obtained from Addgene (Plasmid #79987 and #45461, respectively) [33,35]. To clone miRFP720 [34], the site-directed mutagenesis was performed using the iRFP720 as a template and the following primers: FW-ATGGCCGCCAAGCC TGCAAGCGAGTCGCCCAGGTTCTGG/RV-CCAGAACCTGGGCGACTCGCTTGCAGGCTTGGCGGCCAT, FW-TG CAAGCGAGTCGCCGAGAGACTGGCCTGGCAGATCGGC/RV-GCCGATCTGCCAGGCCAGTCTCTCGGCGACTCGCTTGCA and FW-GTCGCCGAGAGACTGGCCACGCAG ATCGGCGTGATGGAA/RV-TTCCATCACGCCGATCTGC GTGGCCAGTCTCTCGGCGAC. The primers for the mutagenesis include neither start/stop codons nor restriction enzyme sites. To develop the C99 720-670 biosensor, Turquoise-GL and YPet cDNA in the C99 Y-T biosensor [21] were substituted with that of miRFP670 and miRFP720, respectively. The MGH DNA core was used to verify the sequence of C99 720-670 biosensor.

### 2.2. Cell Culture and Transfection

Chinese hamster ovary (CHO) cells, obtained from ATCC (American Type Culture Collection, Manassas, VA, USA), were maintained in Opti-MEM Reduced Serum Medium (Thermo Fisher Scientific, Waltham, MA, USA) supplemented with 5% FBS (Atlanta Biologicals Inc, Flowery Branch, GA, USA). The cells were authenticated using STR profiling and monitored for mycoplasma contamination every two months. Lipofectamine 3000 (Thermo Fisher Scientific, Waltham, MA, USA) was used for transient transfection according to the manufacturer’s instructions.

### 2.3. Subcellular Fractionation

The cytosolic and membrane fractions from cells were purified using Subcellular Protein Fractionation Kit for Cultured Cells (Thermo Fisher Scientific, Waltham, MA, USA) according to the manufacture’s protocol. The successful purification of membrane and cytosolic fractions was verified by the detection of Na^+^/K^+^-ATPase (MilliporeSigma, Burlington, MA, USA) and β-tubulin (Cell Signaling Technology, Danvers, MA, USA), respectively.

### 2.4. Western Blotting

The cells were lysed in a cell lysis buffer (1% Triton X-100, 0.25% NP-40, 10 mM Tris, 2 mM EDTA, 150 mM NaCl, pH 7.4) with protease and phosphatase inhibitor cocktail (Thermo Fisher Scientific, Waltham, MA, USA). Thirty minutes post incubation on ice, each sample was centrifuged, and the supernatants were collected. Protein concentrations were determined using a Pierce BCA Protein Assay Kit (Thermo Fisher Scientific, Waltham, MA, USA). The concentration-normalized samples were diluted in NuPAGE™ LDS Sample Buffer and NuPAGE™ Sample Reducing Agent (Thermo Fisher Scientific, Waltham, MA, USA). After boiling, the samples were subjected to SDS–PAGE on NuPAGE™ 4–12% Bis-Tris Protein gels (Thermo Fisher Scientific, Waltham, MA, USA), followed by transferring to nitrocellulose membranes (Thermo Fisher Scientific, Waltham, MA, USA) using the Bio-Rad Wet electroblotting system (Bio-Rad, Hercules, CA, USA). The detection was performed by immunoblotting with specific primary and corresponding fluorophore conjugated secondary antibodies, and developing the membranes using the digital imaging system LI-COR Odyssey CLx scanner (LI-COR Biosciences, Lincoln, NE, USA). The anti-6E10 (β-Amyloid 1-16) antibody was obtained from BioLegend (San Diego, CA, USA), anti-FLAG antibody from FUJIFILM Wako Pure Chemical Corporation (Osaka, Japan), anti-HA antibody from Abcam (Cambridge, MA, USA), anti-NICD (cleaved Notch1 Val1744) from Cell Signaling Technology (Danvers, MA, USA) and anti-β-actin antibody from Sigma-Aldrich (St. Louis, MO, USA). γ-Secretase inhibitor DAPT, its vehicle control Dimethyl sulfoxide (DMSO) and cycloheximide (CHX) were obtained from Sigma-Aldrich (St. Louis, MO, USA).

### 2.5. Aβ ELISA

The conditioned medium of cells was collected, centrifuged for 5 min at 600 *g*, and the supernatant was diluted and used to measure human Aβ40 and Aβ42 levels. The Human β Amyloid (1-40) ELISA Kit Wako II and Human β Amyloid (1-42) ELISA Kit Wako were used for the measurement according to the manufacture’s protocol (FUJIFILM Wako Pure Chemical Corporation, Osaka, Japan).

### 2.6. LDH Cytotoxicity Assay

The cytotoxicity was determined using the Roche cytotoxicity detection kit (LDH) (Sigma-Aldrich, St. Louis, MO, USA) according to the manufacturer’s protocol. Briefly, 50 μL of the conditioned medium were mixed with 50 μL of the reaction mixture and incubated in the dark for 20 min at room temperature. The absorbance at 490 nm was read using the Wallac 1420 Victor2 Multilabel Microplate Reader (PerkinElmer, Waltham, MA, USA).

### 2.7. Spectral FRET Analysis

Lasers at wavelengths of 405 and 640 nm were used to excite Turquoise-GL in the C99 Y-T and miRFP670 in the C99 720-670 biosensors, respectively. The emitted fluorescence from the donors (Turquoise-GL or miRFP670) and the acceptors (YPet or miRFP720) was detected at 470 ± 10 (Turquoise-GL), 530 ± 10 (YPet), 670 ± 10 (miRFP670) and 710 ± 10 nm (miRFP720) using the Lambda scanning mode on an Olympus FV3000RS Confocal Laser Scanning Microscope equipped with CO_2_/heating units (Tokai-Hit STX-Co2 Digital CO2 Gas Mixing System, STFX model). A 10×/0.25 NA objective was used for the imaging. The Olympus Z drift compensation module (TruFocus) was used to maintain focus during time-lapse imaging. Average pixel fluorescence intensity for the whole cell after subtraction of the background fluorescence was measured using Image J. The emission intensity of YPet over that of Turquoise-GL (Y/T) and the miRFP720 emission over that of miRFP670 (720/670) ratios were used as readouts of the FRET efficiency, which reflect the relative proximity between the donor and acceptor. Pseudo-colored images were generated in MATLAB (MathWorks, Natick, MA, USA).

### 2.8. Statistics

Statistical analysis was performed using GraphPad Prism 8 (GraphPad Software, San Diego, CA, USA). To determine the Gaussian distribution of the data and the variance equality, the D’Agostino and Pearson omnibus normality test was applied. A standard unpaired Student’s *t*-test, Mann–Whitney U test, one-way factorial ANOVA or Repeated Measures ANOVA followed by Bonferroni’s post-hoc analysis was applied to compare the data. The Pearson correlation coefficient was applied to measure linear correlation. A *p*-value of <0.05 was considered a predetermined threshold for statistical significance. All values are given as means ± SD. All experiments were repeated in at least three independent trials, and the number of biological replicates in an experiment is shown. The calculation of sample size was based on previous results from our laboratory and power calculations [21]. Briefly, we used the C99 Y-T FRET biosensor, which has approximately 20–25% dynamic range and needed to have approximately 30 cells/group to reach statistical difference in the spectral FRET analysis.

## 3. Results

### 3.1. The C99 720-670 Biosensor Is Cleaved by Endogenous PS/γ-Secretase

**Figure 1:**
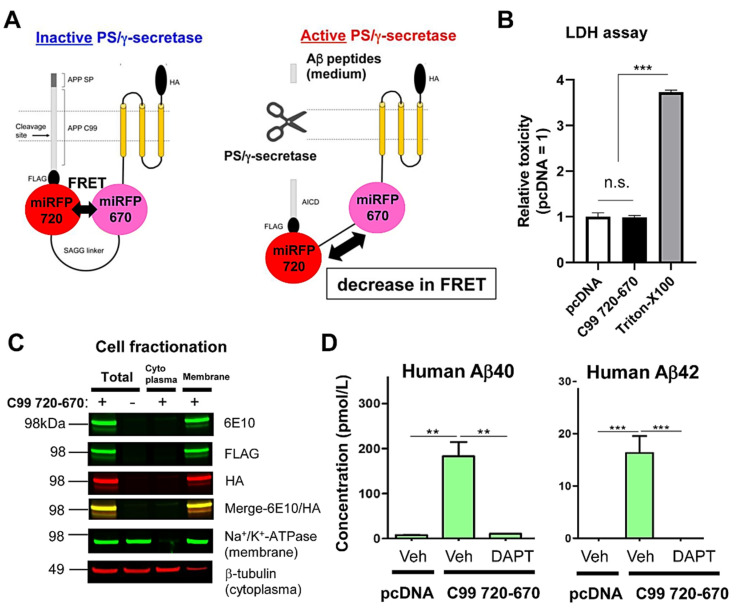
The C99 720-670 biosensor is cleaved by PS/γ-secretase. (**A**) A schematic diagram of the C99 720-670 biosensor. Endogenous PS/γ-secretase cleaves the APP C99 in the C99 720-670 biosensor, which results in a decrease in FRET between miRFP670 (donor) and miRFP720 (acceptor). (**B**) The LDH cytotoxicity assay revealed that the C99 720-670 biosensor causes no toxicity in CHO cells. The 1% Triton-100X treatment was used as a positive control of toxicity. *n* = 4 biological replicates; mean ± SD; n.s., not significant; *** *p* < 0.001, one-way factorial ANOVA. (**C**) The cell fractionation assay evidenced the integration of C99 720-670 biosensor in membrane. The 6E10 antibody recognizes the N-terminus, and the FLAG and HA antibodies capture the middle and the C-terminus of C99 720-670 biosensor, respectively. Na^+^/K^+^-ATPase and β-tubulin were used as membrane and cytoplasmic markers, respectively. (**D**) Detection of human Aβ40 (**left**) and Aβ42 (**right**) in the conditioned medium of C99 720-670 expressing CHO cells by ELISA, which was inhibited by the treatment with 1 µM DAPT (PS/γ-secretase inhibitor). *n* = 3 biological replicates; mean ± SD; ** *p* < 0.01, *** *p* < 0.001, one-way factorial ANOVA.

To better understand the functional relationship between PS/γ-secretase activity and other cellular signaling pathway(s) in real-time, a new red-shifted biosensor reporting PS/γ-secretase activity that is spectrally compatible with the biosensors for monitoring other essential cellular events needs to be developed. This study reports developing and validating a novel PS/γ-secretase activity biosensor C99 720-670 probe utilizing NIR fluorescent proteins: miRFP670 [33] and miRFP720 [34] (Figure 1A). For the development of the C99 720-670 biosensor, Turquoise-GL and YPet in our recently reported C99 Y-T biosensor [21] were replaced with miRFP670 and miRFP720, respectively. First, to examine if the C99 720-670 biosensor is nontoxic in cells, we expressed the C99 720-670 biosensor or empty vector in CHO cells, followed by performing the LDH cytotoxicity assay. We used CHO cells in our present study because the line is well known to express endogenous functional PS/γ-secretase [36,37] and is widely used to examine its role. We found that LDH level in the conditioned medium of the C99 720-670 biosensor expressing CHO cells was similar to that in the cells expressing empty vector, indicating that the C99 720-670 biosensor does not cause toxicity in cells (Figure 1B). A cell fractionation assay verified that the C99 720-670 biosensor is integrated into the membrane (Figure 1C). To determine if the C99 720-670 biosensor is cleaved by endogenous PS/γ-secretase, we expressed the C99 720-670 biosensor or empty vector in CHO cells, and the cells were treated with γ-secretase inhibitor (GSI), DAPT or vehicle control for 16 h. The conditioned medium was then collected to measure “human” Aβ40 and Aβ42 by ELISA, which are the products of C99 720-670 biosensor cleavage by PS/γ-secretase. Expression of the C99 720-670 biosensor allowed clear detection of human Aβ40 and Aβ42 in the conditioned medium at approximately 10:1 ratio of Aβ40 to Aβ42, which was blocked by DAPT treatment (Figure 1D). These results indicate that endogenous PS/γ-secretase processes the C99 720-670 biosensor in CHO cells.

### 3.2. The C99 720-670 Biosensor Reports Endogenous PS/γ-Secretase Activity in Live Cells

Next, we asked if PS/γ-secretase-mediated processing of the C99 720-670 biosensor results in a change in FRET between the donor and acceptor fluorophores, which would indicate the ability of the probe to report endogenous PS/γ-secretase activity in live cells. To do so, we first examined the spectral properties of fluorescence emission in CHO cells expressing miRFP670, miRFP720 or the C99 720-670 biosensor. The cells were excited with a 640 nm wavelength laser, and the emitted fluorescence was detected from 650 to 730 nm using 20 nm bandwidth channels. As expected, in cells expressing miRFP670 or miRFP720, the emission peak was detected in the 670 ± 10 nm channel or the 710 ± 10 nm channel, respectively (Supplementary Materials Figure S1A,B). We found that 640 nm excitation with equal laser power produced 1.6 times higher emission intensity in the 670 ± 10 nm channel of the miRFP670 expressing cells than in the 710 ± 10 nm channel of miRFP720-positive cells (Figure S1A,B). On the other hand, the cells expressing the C99 720-670 biosensor revealed 2.1 times higher emission intensity in the 710 ± 10 nm channel compared to that in the 670 ± 10 nm channel (Figure S1C), suggesting that this increase in the miRFP720 emission is due to FRET from the miRFP670 donor to miRFP720 acceptor in the C99 720-670 biosensor expressing cells.

**Figure 2:**
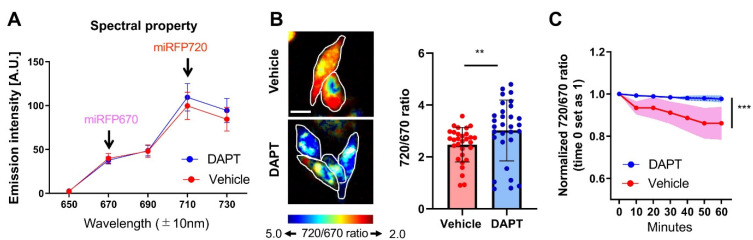
Processing of the C99 720-670 biosensor by PS/γ-secretase decreases the FRET between miRFP670 and miRFP720. (**A**) Spectral property of the CHO cells expressing the C99 720-670 biosensor. The cells were excited by a 640 nm laser, and fluorescence emission intensity within 650–730 ± 10 nm was measured. One micromolar DAPT decreased the 670 ± 10 nm but increased the 710 ± 10 nm emission. *n* = 30 cells. (**B**) The pseudo-colored images of the 710 ± 10 over 670 ± 10 nm emission ratio (720/670 ratio) in the CHO cells corresponding to Figure 2A. Scale bar, 50 um. The 720/670 ratio was significantly increased in the cells treated with 1 µM DAPT compared to vehicle control. *n* = 30 cells; mean ± SD; ** *p* < 0.01, Mann–Whitney U test. (**C**) Longitudinal monitoring of the 720/670 ratio in the C99 720-670 biosensor expressing cells. ROIs were created around the entire cell. Then, the 720/670 ratio was measured every 10 min for 1 h by time-lapse spectral FRET analysis. The ratio at time point 0 was set as 1, and the relative 720/670 ratio was calculated on a cell-by-cell basis. A time-dependent decrease in the 720/670 ratio in vehicle-treated cells was cancelled by the treatment with 1 µM DAPT (*n* = 20 cells). mean ± SD; *** *p* < 0.001, Repeated Measures ANOVA.

Then, CHO cells expressing the C99 720-670 biosensor were treated with either vehicle control or DAPT to block PS/γ-secretase activity. We found that the emission fluorescence intensity increased in the 710 ± 10 nm channel and slightly decreased in the 670 ± 10 nm channel in DAPT treated cells compared to the cells with vehicle treatment (Figure 2A). This results in a statistically significant increase in the emission ratio of miRFP720 over miRFP670 (720/670 ratio) in the DAPT treated cells compared to that in the vehicle-treated cells (Figure 2B). To ensure that the increased 720/670 FRET ratio does not come from a higher expression level of the C99 720-670 biosensor, we performed a correlation analysis between the 720/670 ratio and the miRFP670 donor fluorescence emission intensity, which reflects the expression level of C99 720-670 sensor in each cell. We found that there was no positive correlation between the 720/670 ratio and the expression level of C99 720-670 biosensor (Figure S2), indicating that the difference in the FRET ratio is not dependent on the level of the probe but rather on the change in proximity and/or orientation between miRFP670 and miRFP720 due to changes in PS/γ-secretase activity.

We further examined if the C99 720-670 biosensor can longitudinally report PS/γ-secretase activity. The cells expressing C99 720-670 biosensor were pre-treated with either vehicle or DAPT. Then, the cells were treated with cycloheximide (CHX), and the 720/670 ratio was monitored every 10 min for 1 h post CHX treatment in individual cells. CHX prevents the de-novo synthesis of both the biosensor (substrate) and PS/γ-secretase (enzyme), thus allowing interpretation of the changes in 720/670 emission ratio as the processing efficiency of the C99 720-670 biosensor by PS/γ-secretase. We found a time-dependent decrease in the 720/670 ratio in vehicle treated cells compared to DAPT, suggesting that the C99 720-670 biosensor was processed by endogenous PS/γ-secretase over time (Figure 2C). Thus, we conclude that a lower 720/670 ratio is associated with high PS/γ-secretase processing activity, and hence the cleavage of C99 720-670 biosensor by endogenous PS/γ-secretase could be reported by monitoring a decrease in FRET between miRFP670 and miRFP720.

### 3.3. The C99 720-670 Biosensor is Compatible with CFP-YFP Based Biosensors

**Figure 3:**
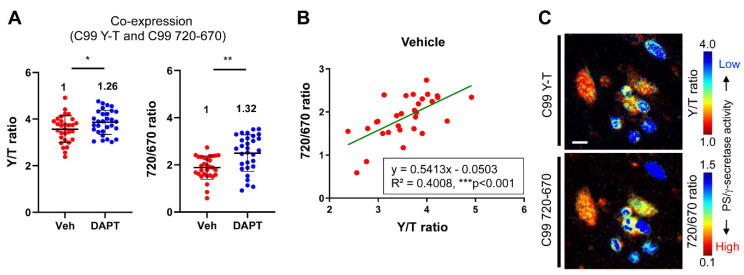
The C99 720-670 biosensor is compatible with the C99 Y-T biosensor. (**A**) The C99 720-670 biosensor was co-transfected with the C99 Y-T biosensor into CHO cells, followed by the treatment with 1 µM DAPT or vehicle control. The cells were excited with 405 and 640 nm lasers, and the emissions of 470, 530, 670 and 710 nm (±10 nm) were measured. The emission of 530 over 470 nm (Y/T ratio) and 710 over 670 nm (720/670 ratio) were significantly increased by DAPT treatment compared to vehicle control. The acceptor over donor emission ratio in cells treated with DAPT is divided by that in corresponding vehicle-treated cells, and the fold change compared with the C99 Y-T biosensor is shown (the vehicle condition set as 1). *n* = 30 cells; mean ± SD; * *p* < 0.05, ** *p* < 0.01, Mann–Whitney U test. (**B**) Scatter plots represent the Y/T ratio and 720/670 ratio in the same cells in vehicle treated condition (corresponding to the Figure 3A). The 720/670 ratio is correlated with the Y/T ratio. Y = 0.5413X − 0.0503, R^2^ = 0.4008, *** *p* < 0.001, Pearson correlation coefficient. (**C**) The pseudo-colored images of the Y/T ratio (top panel) and 720/670 ratio (bottom) in the same cells. Scale bar, 50 um.

To further validate the C99 720-670 biosensor’s compatibility with biosensors that range in blue-yellow color, and its suitability for multiplexed imaging, we co-expressed the C99 720-670 and C99 Y-T biosensors in CHO cells. Treatment of the cells with DAPT was used to block PS/γ-secretase activity. The cells expressing both the C99 720-670 and C99 Y-T biosensors were excited by 405 and 640 nm wavelength lasers, and emitted fluorescence was detected in the channels of 470 ± 10 (emission peak of Turquoise-GL), 530 ± 10 (YPet), 670 ± 10 (miRFP670) and 710 ± 10 nm (miRFP720). A region of interest (ROI) was created on multiple cells to measure the fluorescence intensity in the four emission channels on a cell-by-cell basis. We found a statistically significant increase in both the 720/670 ratio and the ratio of YPet over Turquoise-GL emission (Y/T ratio) in the DAPT treated cells, compared to that in the vehicle-treated cells (Figure 3A). Overall, the cells reporting higher Y/T ratios reveal higher 720/670 ratios, and this trend is the same for the cells with lower ratios in vehicle treated condition (Figure 3B). This finding was further verified by comparing the pseudo-colored images of the Y/T and the 720/670 ratios in the same cells (Figure 3C). These data implicate that PS/γ-secretase can be simultaneously measured by the C99 720-670 and C99 Y-T biosensors and evidence the compatibility of the C99 720-670 biosensor with CFP-YFP based FRET biosensors.

## 4. Discussion

PS/γ-secretase plays a pivotal role in both development [14,15] and numerous diseases [38]. To determine how PS/γ-secretase activity is spatiotemporally regulated within the normal and pathological environment of cells, we recently engineered a FRET-based biosensor, the so-called C99 R-G biosensor, that contains an APP-based immediate PS/γ-secretase substrate (i.e., APP C99) and EGFP and RFP as the donor and acceptor fluorophores, respectively [21]. Replacement of the donor EGFP and acceptor RFP with Turquoise-GL and YPet, respectively, and modification of the donor/acceptor linker length yielded a higher sensitivity C99 Y-T biosensor. The C99 Y-T biosensor is processed by PS/γ-secretase being similar to C99, suggesting that fusion of the donor/acceptor fluorescent proteins and the anchor domain affect neither the trafficking of C99 nor the accessibility of PS/γ-secretase to the C99 cleavage site. The C99 Y-T biosensor with microscopy-based imaging assays enables quantitative monitoring of PS/γ-secretase activity overtime on a cell-by-cell basis in live cells [21]. However, how molecular and cellular factors spatiotemporally regulate PS/γ-secretase activity, as well as the consequence of changes in PS/γ-secretase activity, is still poorly understood. To answer these crucial questions, the development of new tool(s) that enables simultaneous monitoring of PS/γ-secretase activity and other cellular events in a single cell is required. We present a new FRET biosensor with red-shifted excitation and emission wavelengths that we successfully developed—C99 720-670 biosensor—which contains miRFP670 and miRFP720 fluorescent proteins as FRET donor and acceptor, respectively. Our extensive validation reveals that the C99 720-670 biosensor is processed by endogenous PS/γ-secretase activity (Aβ40: 183.0 ± 55.2 (pM) (vehicle) vs. 10.7 ± 0.3 (DAPT), ** *p* < 0.01, Aβ42: 16.3 ± 5.5 (vehicle) vs. under detection range (0.0 ± 0.0) (DAPT), ***p < 0.001) (Figure 1), reports its activity in live cells (the 720/670 ratio: 2.47 ± 0.66 (vehicle) vs. 3.02 ± 1.17 (DAPT), ** *p* < 0.01) (Figure 2) and, importantly, is spectrally compatible with a CFP-YFP based FRET biosensor (y = 0.5413x − 0.0503, R**^2^** = 0.4008, *** *p* < 0.001) (Figure 3).

Increasing numbers of NIR fluorescent proteins, such as miRFP670, have been engineered from bacterial phytochrome photoreceptors (BphP) [33,34,35,39,40]. The NIR fluorescence of BphP-based fluorescent proteins is derived from the incorporation of biliverdin IXa that is the most red-shifted natural chromophore [41]. miRFP720 is the most red-shifted fluorescent protein recently engineered, offering miRFP670 and miRFP720 as a new donor–acceptor pair for the development of NIR FRET biosensor [34]. This new FRET pair possesses three crucial characteristics for FRET imaging applications: effective brightness, monomeric state and distinct spectral property. The first NIR FRET biosensor utilizing miRFP670 and miRFP720 was developed for monitoring Rac1 GTPase activity [34]. We built on this discovery to generate the C99 720-670 biosensor for quantitative monitoring of PS/γ-secretase activity and show that the C99 720-670 biosensor displays similar sensitivity (the relative change between vehicle treated cells and DAPT: 32%) as the C99 Y-T probe (26%) (Figure 3).

The utility of the C99 720-670 biosensor derives from the spectral properties of miRFP670 and miRFP720 that enable multiplexed imaging with visible fluorophores. The compatibility between the C99 720-670 biosensor and CFP-YFP based biosensors is evidenced by the fact that the C99 720-670 biosensor and the previously developed C99 Y-T biosensor can report PS/γ-secretase activity in the same cell (y = 0.5413x − 0.0503, R**^2^** = 0.4008, *** *p* < 0.001) (Figure 3). Thus, the spectral compatibility between the C99 720-670 and CFP-YFP based biosensors will allow simultaneous monitoring of PS/γ-secretase activity and other essential molecular/cellular events in a single cell. This ability will help elucidate both upstream modulators of the PS/γ-secretase activity and outcomes downstream of altered PS/γ-secretase activity. Post-translational modification, phosphorylation, in particular, represents a common mechanism of regulating protein conformation and activity. Of note, multiple phosphorylation sites are identified in PS, the catalytic component of γ-secretase, and the phosphorylation on some of these sites is shown to affect the stability [26,28,29], conformation [31] or localization of PS/γ-secretase [42]. These studies implicate that PS1/2 phosphorylation could be one of the regulators of γ-secretase activity. FRET-based biosensors using yellow to blue spectra fluorophore have been developed to monitor protein kinases [43,44,45,46]. Multiplexed live cell imaging using such kinase biosensors and the C99 720-670 probe would help uncover how “naturally” up- or downregulated kinase activity impacts PS/γ-secretase activity in a living cell. Furthermore, an increasing number of optogenetic tools for regulating the activity of protein kinases have been developed [47,48]. A combination of the C99 720-670 biosensor with these optogenetic tools would reveal how changes in kinase activity spatiotemporally affect PS/γ-secretase activity in individual cells.

Several orange-red shifted fluorescent proteins have been used to produce FRET biosensors, and their compatibility with CFP-YFP based biosensors is validated [49,50,51]. However, we chose miRFP670 and miRFP720 as the donor and acceptor in the present study since emitted light from these fluorescent proteins would more efficiently penetrate deep tissue, considering light scattering and light absorption by hemoglobin, water and other intrinsic chromophores are at a minimal level, and autofluorescence is low in the NIR region of the spectrum [52,53,54]. This would allow successful monitoring of PS/γ-secretase activity and multiplexed imaging in deep tissue *in vivo*. Importantly, the suitability of NIR fluorescent proteins in deep brain imaging and their compatibility with the green-to-yellow range fluorescent proteins has been validated [55], supporting the feasibility of multiplexed imaging *in vivo*. These unique properties will enable a better understanding of the regulation and consequences of PS/γ-secretase in more physiological conditions, such as in intact cells/tissues in live mouse models.

Over 300 missense mutations that result in autosomal dominant familial AD are identified on PS genes (http://www.alzforum.org/mutations). As expected from the fact that these mutation sites are scattered over the entire sequence of PS genes, a comprehensive analysis verifies that familial AD mutations overall cause loss of PS/γ-secretase activity, with some of these mutations being “partial” and others “complete” loss of activity [56]. However, the exact molecular mechanism(s) by which PS missense mutations cause familial AD and whether similar alterations in PS/γ-secretase lead to sporadic AD neurodegeneration remain unclear. The study in which familial AD mutant PS1 knock-in mice were for the first time generated showed that primary neurons cultured from the M146V PS1 knock-in mice and treated with excess glutamate reveal greater neuronal vulnerability [57]. As one of the mechanisms by which M146V PS1 knock-in leads to neuronal vulnerability, abnormal calcium regulation is proposed (e.g., increased calcium influx and calcium overload post glutamate treatment) [57]. Since the development and use of imaging tools for quantitative monitoring of toxicity linked events such as calcium dysregulation [58,59] or oxidative stress [60], PS/γ-secretase activity could be longitudinally monitored along with the toxicity-linked events in live neurons. Such multiplexed imaging may shed light on the mechanistic relationship between PS/γ-secretase and neuronal vulnerability.

## 5. Conclusions

We developed and validated the novel NIR FRET biosensor C99 720-670, which enables reporting endogenous PS/γ-secretase activity in live cells. The C99 720-670 biosensor is compatible with CFP-YFP based biosensor(s), which will provide unique opportunities for monitoring PS/γ-secretase activity along with other molecular/cellular factors, in addition to using optogenetic tools, and in deep tissue in vivo. The C99 720-670 biosensor could open new avenues to better understand the dynamic nature of the PS/γ-secretase and explore the regulation and consequences of altered PS/γ-secretase activity.

## Supplementary Materials

The following are available online at https://www.mdpi.com/1424-8220/20/21/5980/s1, Figure S1: Emission fluorescence spectral properties of cells expressing miRFP670, miRFP720 or the C99 720-670 biosensor; Figure S2: Correlation between the 720/670 ratio and C99 720-670 probe expression level.
