# Supplementary material for: A Novel NIR-FRET Biosensor for Reporting PS/γ-Secretase Activity in Live Cells
Source: Sensors (Basel). 2020 Oct 22;20(21):5980. doi: 10.3390/s20215980 (PMC7660074; doi:10.3390/s20215980)
Supplement: Supplementary file 1 [file sensors-20-05980-s001.pdf]

Supporting information

A novel NIR-FRET biosensor for reporting PS/γ-secretase activity in live cells

Mei CQ Houser, Steven S Hou, Florian Perrin, Yuliia Turchyna, Brian J Bacskai, Oksana Berezovska, Masato Maesako*

Alzheimer Research Unit, MassGeneral Institute for Neurodegenerative Disease, Massachusetts General Hospital, Harvard Medical School, 114, 16th street, Charlestown, MA 02129

***Corresponding Author**

Masato Maesako, PhD (MMAESAKO@mgh.harvard.edu)

Supplemental Figure 1

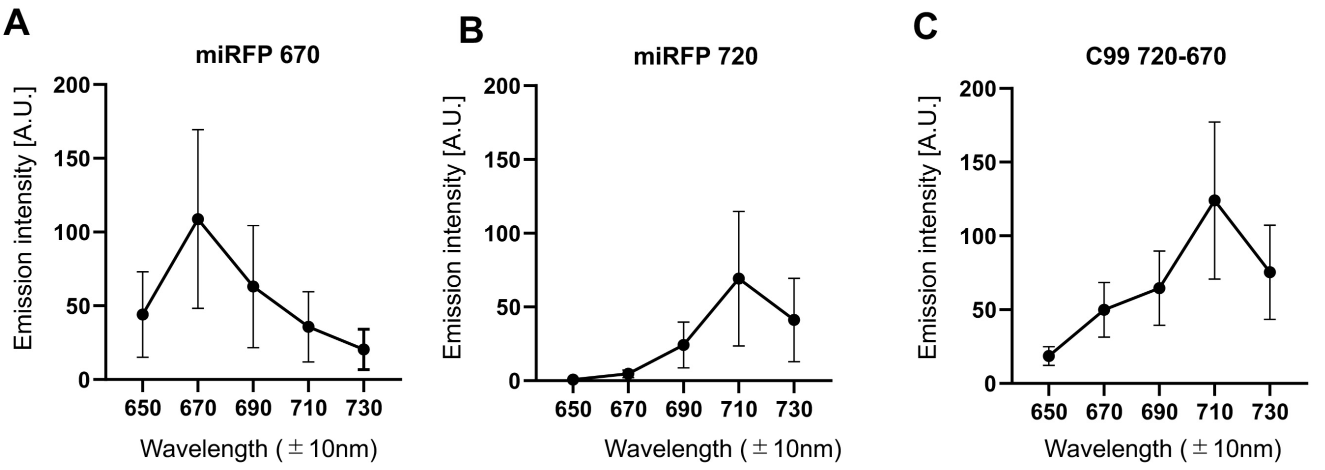

Figure S1. Emission fluorescence spectral properties: CHO cells expressing miRFP670 (A), miRFP720 (B), or the C99 720-670 biosensor (C) were excited by a 640nm laser. Fluorescence emission intensity within 650-730±10nm was measured (n = 30 cells). Mean ± SD

Supplemental Figure 2

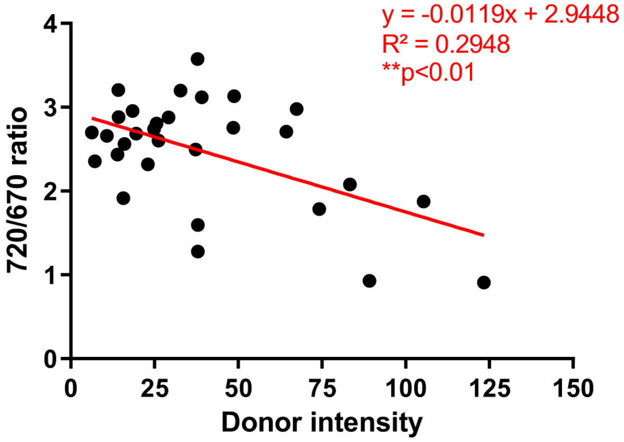

Figure S2. Correlation between the 720/670 ratio and C99 720-670 probe expression level: Correlation analysis between the 720/670 ratio (corresponding to Fig. 2A and 2B) and miRFP670 donor emission intensity (which reflects the biosensor expression level in the cell) shows a negative correlation. This indicates that higher 720/670 ratio does not come from higher biosensor expression. n = 30 cells, Pearson correlation coefficient, $y = -0.0119x + 2.9448$, $R^2 = 0.2948$, $p = 0.0019$
